# Long-Term Electrode–Skin Impedance Variation for Electromyographic Measurements

**DOI:** 10.3390/s23208582

**Published:** 2023-10-19

**Authors:** Andreia S. P. Sousa, Andreia Noites, Rui Vilarinho, Rubim Santos

**Affiliations:** 1 Center for Rehabilitation Research—Human Movement System (Re)habilitation Area, School of Health, Polytechnic of Porto, Rua Dr. António Bernardino de Almeida, 400, 4200-072 Porto, Portugal; arn@ess.ipp.pt (A.N.); rvilarinho@ufp.edu.pt (R.V.); rss@ess.ipp.pt (R.S.); 2 FP-I3ID, Escola Superior de Saúde-Fernando Pessoa, 4200-253 Porto, Portugal

**Keywords:** electrode–skin impedance, electromyography, stability

## Abstract

This study aims to observe the evolution of the electrode–skin interface impedance of surface EMG electrodes over the time taken to determine the time of stabilization. Eight healthy subjects participated in the study. Electrode–skin impedance was evaluated in the rectus abdominal muscle every five minutes, over a total period of 50 min. A reduction of 13.23% in the impedance values was observed in minute 10 (*p* = 0.007), and a reduction of 9.02% was observed in minute 15 (*p* = 0.029). No statistically significant differences were observed in the other instants evaluated. The findings obtained in the present study demonstrate a decrease in electrode–skin impedance from minute 5 to minute 15, followed by a stabilization period with a low percentage of variation till minute 50.

## 1. Introduction

Surface electromyography (EMG) is a convenient index of muscle excitation and allows for a description of muscular patterns [1]. However, absolute EMG amplitude values are not reliable due to many factors [2]. The variance of the estimate can be substantially reduced with special techniques, such as signal whitening and multichannel processing [3]. Nevertheless, the main limitation in the interpretation of EMG amplitude results not from processing algorithms but from the masking effects of unwanted factors. Recordings of surface EMG often contain a substantial noise component. This noise signal can severely impair the resolution of surface EMG recordings [4,5] and lead to waveform distortion and power line interference in the recorded EMG [5]. When muscle activation is at a very low level, an acceptable signal-to-noise ratio can only be obtained by markedly reducing the noise level. A potentially large source of noise is that caused by the electrode–skin interface [6,7,8]. This problem can be reduced by minimizing the electrode–skin impedance [5].

Traditionally, surface EMG electrodes have been applied to the skin after rigorous preparation, which has included shaving the electrode site, rubbing with gel, abrasion with sandpaper, and cleaning with alcohol to reduce the skin impedance to below 10 kΩ [7,9,10,11]. Values below 10 kΩ have been described to result in a good EMG signal [12]. However, these values are not stable for long. It has been demonstrated that there is a reduction of 20 to 30% in impedance values during the first 5 min after electrode placement [13] and of 50% in the first 20 min after its application when metals were placed face-to-face with electrodes [14]. This assumes special relevance because the stability of impedance over time and the balance of impedance between electrode sites have a considerable effect on the signal-to-noise ratio of the measured EMG signal [7], both in terms of noise levels and spatial resolution. Therefore, it is very important that the impedance values remain consistent throughout the measurement session. According to our knowledge, the time after electrode placement that the electrode–skin impedance stabilizes has not been determined yet.

This study aims to observe the evolution of the electrode–skin interface impedance of surface EMG electrodes over the time taken to determine the time of stabilization. Knowledge of changes in impedance over time may be of use to improve the reliability of EMG measurements.

## 2. Methods

### 2.1. Subjects

The study included 8 healthy participants between the ages of 18 and 30, 3 males and 5 females (age = 19.8 ± 1.5 years, height = 1.68 ± 0.12 m, weight = 69.9 ± 10.3 kg; mean ± SD). The study was conducted according to the ethical norms of the institutions involved and conformed to the Declaration of Helsinki, with informed consent from all participants. The study is registered in Clinical trials with the reference NCT02110927.

### 2.2. Instrumentation

Self-adhesive silver/silver chloride electromyographic pre-gelled (wet gel) electrodes were used in a bipolar configuration with a distance of 20 mm between detection surface centres (Noraxon Dual Electrods, Noraxon USA, Inc.). Silver/silver chloride electrodes are generally assumed to have a more stable contact potential and, consequently, better noise behaviour than other metals when brought into contact with an electrolyte [14]. Moreover, wet-gel electrodes are associated with lower noise compared to hydrogel electrodes [14]. Electrode–skin impedance was assessed as the opposition to the flow of a small alternating current between electrodes (two) [15,16] and was performed using a digital multimeter UniVolt DT81RS model (DIETZEL GmbH, Vienna, Austria) with an impedance level of 10 MΩ, a bandwidth of 45–400 Hz, and a commercial electrode impedance checker (Noraxon USA, Inc.).

### 2.3. Procedures

The skin surface of the subjects’ rectus abdominal muscle was prepared to reduce electrical resistance to less than 10 kΩ (confirmed by a commercial electrode impedance checker) by shaving the electrode site, rubbing with gel, and cleaning with alcohol. We selected this muscle since this study is part of a larger study that aims to evaluate the influence of microcurrent therapy together with exercise on the reduction of abdominal adiposity and on the degree of rectus abdominal muscle activation. An electrode was placed 3 cm to the right of the umbilicus. An interval of 5 min was established between the electrode positioning and the beginning of measurement.

Subjects were positioned in supine positions and were instructed to relax for 50 min. Impedance values were collected every 5 min, specifically in minutes 5, 10, 15, 20, 25, 30, 35, 40, 45 and 50 after electrode placement. Because the commercial electrode impedance checker presents values in intervals, a multimeter was used to assess the absolute values of electrode–skin impedance. In minute 5, after electrode placement, impedance was measured with both the multimeter and the commercial electrode impedance checker. With this procedure, we guaranteed that in the beginning of the evaluation, all participants presented an electrode–skin impedance value lower than 2 kΩ, as measured with the commercial electrode impedance checker, and at the same time functioned as a reference to confirm the data obtained with the multimeter. As absolute values of electrode–skin impedance values cannot be assessed with the commercial impedance checker, the results obtained in the present study are obtained with the multimeter.

### 2.4. Statistics

The collected data were analyzed using the software Statistic Package Social Science version 28 (SPSS) from IBM Company (Armonk, NY, USA). Upon verification of the underlying assumptions, normality (Shapiro–Wilk test and histogram analysis), and sphericity (Mauchly’s test of sphericity), a repeated-measures analysis of variance (ANOVA) was used with a 5% significance level to compare impedance values over the time period. The Least Significant Difference (LSD) test was used for the post hoc analyses.

## 3. Results

**Figure 1:**
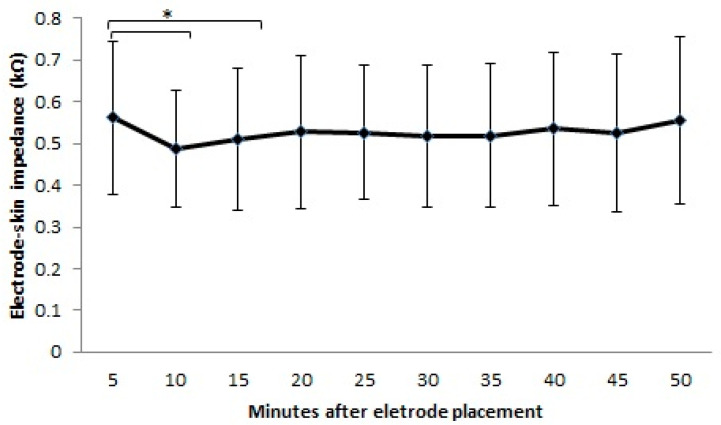
Mean and standard deviation values of electrode–skin impedance measured in minute 5, minute 10, minute 15, minute 20, minute 25, minute 30, minute 35, minute 40, minute 45, and minute 50 after electrode placement. Statistical significant differences are identified with an *.

**Figure 2:**
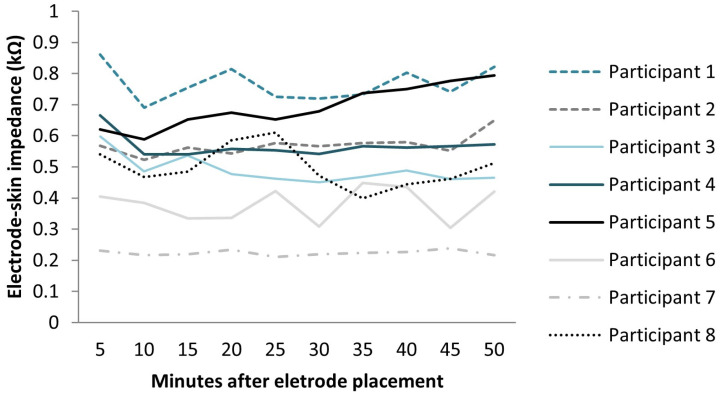
Electrode–skin impedance measured in each participant in minute 5, minute 10, minute 15, minute 20, minute 25, minute 30, minute 35, minute 40, minute 45 and minute 50 after electrode placement.

From minute 5 to minute 50, the mean values of electrode–skin impedance ranged from 0.49 to 0.56 kΩ (Figure 1). When considering the values presented by each participant, the electrode–skin impedance values ranged from 0.86 to 0.24 kΩ (Figure 2). Statistically significant differences were observed between the intervals measured (F = 75.209; df = 9; ES = 0.915; *p* < 0.0001) with an observed power of 0.841. The higher variation occurred from minute 5 (0.56 kΩ) to minute 10 (0.49 kΩ) and to minute 15 (0.51 kΩ). The impedance value after 10 min and 15 min was reduced by 12.3% (*p* = 0.007) and 9.02% (*p* = 0.029), respectively, compared with that after 5 min after electrode placement. As can be observed in Figure 1, there was a tendency for the impedance values to increase till minute 50, taking as reference the value obtained at minute 15, but no significant differences were observed in this interval. In fact, a small variation in the impedance values was observed from minute 15 to minute 50, with an averaged variation of 2.9%.

## 4. Discussion

The electrode–skin interface generates a D/C voltage potential, mainly caused by a large increase in impedance from the outermost layer of skin, including dead skin material and oil secretions [15,17]. This D/C potential, common to all electrodes, can be minimized with proper skin preparation [17]. In the present study, decreased values of electrode–skin impedance (0.487–0.561 kΩ) were obtained during the period evaluated after skin preparation, probably due to this procedure but also due to a saturation of the upper skin layers with electrolyte [6,14]. This phenomenon seems to also explain the differences obtained in electrode–skin impedance over the period evaluated.

The results of the present study demonstrate decreased impedance values in minutes 10 and 15 compared to minute 5 after electrode placement. The slow penetration of the gel into the skin, the wrinkling of the moist skin resulting in an increased contact area [14], and the hydration of dead skin cells by the electrode gel [18] seem to be the mechanisms that explain the decrease in the impedance values [19,20,21]. In fact, when pre-gelled electrodes are applied to alcohol-wiped skin, the gel will eventually penetrate the degreased skin more readily once the electrode has been on the skin several minutes [22]. Furthermore, subtle changes in the dynamics of the lipids due to the mobilization and incorporation of cholesterol and long-chain lipid species into the fluid lipid fraction are suggested to occur upon hydration [23]. The magnitude of the electrode–skin impedance reduction found in the present study was lower than that obtained previously for the entire interval of evaluation [13,14]. This may result from the decreased initial impedance values obtained in the present study, as this factor has been demonstrated to affect the magnitude of the reduction over time [12]. In the present study, the initial impedance values, confirmed with the commercial impedance checker, were lower than 2 kΩ. The low values of electrode–skin impedance can be related to the skin preparation procedures adopted in the presented study, as skin abrasion, by removing the insulating barrier presented by the epidermis, favors direct charge transfer between the skin, gel, and the electrode [24]. The values of the initial electrode–skin impedance are in accordance with previous studies [25].

The results of the present study demonstrate that from minute 15 to minute 50, there was an average increase in the electrode–skin impedance values of 2.9%. Considering the evidence demonstrating that below 55 kΩ, the spectrum of the surface electromyographic signal will not be affected [12] and that the variations obtained in the present study are negligible [25], it can be argued that, despite the increased impedance values after minute 15, optimal values of skin impedance were obtained in the whole period of the evaluation. In other words, despite the fact that decreased impedance values were found from minute 5 to minute 15, for the abdominal region, electromyography measurements could possibly be performed in the whole period evaluated from minute 5 to minute 50. However, since impedance variation can interfere with electromyographic signal quality metrics such as the signal-to-noise ratio, future studies evaluating this metric are needed to confirm if the values of the signal-to-noise ratio are also stable from minute 15 to minute 50. This metric is particularly relevant for muscle onset measurements [26].

It is important to note that it is not always possible to decrease the electrode–skin impedance to the values obtained in the present study. Since values lower than 10 kΩ are considered acceptable, future studies are required to confirm if, when other initial impedance values are obtained, similar results are obtained. It also should be noted that, despite the fact that contact impedances are dominated by the impedance of the outer layers of the skin [27], the method used in the present study reflects the impedance of everything between the two electrodes, which includes the impedance between each electrode and the living skin tissue lying right beneath it [15]. Therefore, the results obtained in the present study should be extended only to self-adhesive silver/silver chloride EMG pre-gelled (wet gel) electrodes. Future studies are required to confirm if the same results are obtained with other types of EMG electrodes, such as dry electrodes, that have the potential to overcome the challenges associated with wet electrodes, such as their single-time use, the need for skin preparation, the potential to cause skin irritation, and their lack of suitability for long-term monitoring as the gel dries over time, leading to signal degradation [28]. Because dry electrodes have an unstable electrochemical interface due to the absence of gel, which affects their performance, studies on the electrode–skin impedance variation over time are required. Despite the fact that only eight subjects participated in the present study, the observed power is high. However, since electrode–skin impedance varies between subjects and between body regions, studies involving a more heterogeneous sample and other body regions are required.

## 5. Conclusions

This study aimed to describe the variation in the impedance at the skin–electrode interface for EMG measurements over the time taken to determine the time of stabilization. The findings obtained in the present study demonstrate a decrease in electrode–skin impedance from minute 5 to minute 15, followed by a stabilization period with a low percentage of variation till minute 50.

## Data Availability

Not applicable.
